# A direct comparison of attentional orienting to spatial and temporal positions in visual working memory

**DOI:** 10.3758/s13423-021-01972-3

**Published:** 2021-07-21

**Authors:** Anna Heuer, Martin Rolfs

**Affiliations:** Department of Psychology, Humboldt-Universität zu Berlin, Rudower Chaussee 18, 12489 Berlin, Germany

**Keywords:** visual working memory, spatial attention, temporal attention

## Abstract

Different visual attributes effectively guide attention to specific items in visual working memory (VWM), ensuring that particularly important memory contents are readily available. Predictable temporal structures contribute to this efficient use of VWM: items are prospectively prioritized when they are expected to be needed. Occasionally, however, visual events only gain relevance through their timing after they have passed. We investigated retrospective attentional orienting based on temporal position by directly comparing it with orienting to spatial locations, which is typically considered the most powerful selection mechanism. In a colour-change-detection task, in which items appeared sequentially at different locations, symbolic number cues validly indicated the temporal or spatial location of the upcoming probe item either before encoding (precues; Experiment 1) or during maintenance (retrocues; Experiments 1–3). Temporal and spatial cues were physically identical and only differed in their mapping onto either temporal or spatial positions. Predictive cues yielded cueing benefits (i.e., higher accuracy and shorter reaction times) as compared with neutral cues, with larger benefits for precues than for retrocues. Importantly, spatial and temporal cueing benefits did not differ. Equivalent retrocueing benefits were also observed across different cue-probe intervals and irrespective of whether spatial or temporal position was used as retrieval cue, indicating that items were directly bound to temporal position and not prioritized via a space-based mechanism. These findings show that spatial and temporal properties can be used equally well to flexibly prioritise representations held in VWM and they highlight the functional similarities of space and time in VWM.

**Supplementary Information:**

The online version contains supplementary material available at 10.3758/s13423-021-01972-3.

## Introduction

Adaptive and effective behaviour relies heavily on the ability to maintain visual information over short periods of time. As we move and interact with our environment, visual working memory (VWM) bridges temporal gaps in which relevant information is not available to the senses—for instance, when it is occluded by another object or when we look somewhere else—and it allows us to retain visual details of fleeting events that have passed.

The essential function of VWM, however, stands in contrast to its highly limited capacity (e.g., Luck & Vogel, 2013; Ma et al., 2014). One way that is dealt with is by filtering out irrelevant information at encoding, thus preventing it from gaining access to VWM and consuming capacity (e.g., Jost & Mayr, 2016; Vogel et al., 2005). But even representations already in VWM can be flexibly updated according to changes in task relevance during maintenance. While information that is no longer important can be removed from memory (e.g., Souza et al., 2014; Williams et al., 2013), maintenance in different representational states established by the allocation of attention can reflect more graded differences in relevance (e.g., Gunseli et al., 2015; LaRocque et al., 2014; Stokes et al., 2020; van Moorselaar et al., 2015)—as established, for instance, by the likelihoods of items to be tested, by the requirements of an upcoming action, or by item value (e.g., Allen & Atkinson, 2021; Heuer et al., 2020; Heuer & Schubö, 2018; Ohl & Rolfs, 2017). Research on selective processing during maintenance has been fuelled by the introduction of the retrocue paradigm (Griffin & Nobre, 2003; Landman et al., 2003), in which cues presented during the retention interval of a VWM task indicate one or several item(s) as more likely to be subsequently tested (reviewed in Souza & Oberauer, 2016). Countless variations of retrocue types have been used to demonstrate that different visual attributes guide attention within VWM: spatial location (e.g., Astle et al., 2012; Griffin & Nobre, 2003; Heuer & Schubö, 2016b), nonspatial features such as colour or shape (e.g., Heuer & Schubö, 2016a; Heuer et al., 2016; Kalogeropoulou et al., 2017; Li & Saiki, 2014; Pertzov et al., 2013), object categories (e.g., Lepsien & Nobre, 2007; Lepsien et al., 2011), or entire feature dimensions (e.g., Hajonides et al., 2020; Niklaus et al., 2017).

As we live in a dynamic world, temporal properties of visual events should likewise contribute to an optimal utilization of VWM by tuning attention to representations related to relevant points in time. Recent years have seen an increased interest in how temporal attention shapes visual perception (e.g., Nobre et al., 2007; Nobre & van Ede, 2018; Rohenkohl & Nobre, 2011), revealing its profound impact on different stages of sensory processing. Temporal expectations also influence mnemonic representations: Performance is facilitated when items are probed at expected times, indicating that representations are dynamically prioritized based on when they are expected to be required for on-going behaviour (Jin et al., 2019; van Ede et al., 2017). Such expectations are based on predictable temporal structures, like associations between stimuli and their timing, and modulate VWM in a prospective manner. It remains unclear, however, if memorized items can also be retrospectively prioritized, when they gain relevance based on their timing only after they have been encoded and are no longer in view. A traffic accident, for instance, renders any immediately preceding events critical, although they may initially have been perceived as rather unremarkable. A particularly distinctive temporal attribute is the ordinal position of an item in a sequence. Indeed, temporal position is sometimes used as a retrieval cue in tasks with sequential memory item presentation (e.g., Gayet & Peelen, 2019; Harrison & Tong, 2009) and is thus evidently an effective means to access information—which is a necessary prerequisite for attentional prioritization after encoding.

In this study, we investigated attentional orienting based on temporal position by directly comparing it with orienting based on spatial location. Spatial attention is not only the most extensively studied but typically also considered the most powerful selection mechanism. In three experiments, participants performed a spatiotemporal variant of a colour-change-detection task, in which memory items were presented sequentially and at different locations (see also Heuer & Rolfs, 2021). Symbolic number cues validly indicated the probe item based on its spatial or temporal position either before encoding (precues; Experiment 1) or during maintenance (retrocues; Experiments 1–3). Spatial and temporal cues were physically identical and only differed in their mapping onto space or time. Trials with neutral cues, providing no information about the upcoming probe item, were interleaved and served as a baseline. We expected that both spatial and temporal cues would yield cueing benefits—that is, improved performance with predictive as compared to neutral cues. Moreover, the perfectly matched cues allowed us to establish whether information about temporal position is as effective as spatial information in guiding attention to particularly important memory representations.

## Experiment 1

In a first step, we examined the efficacy of fully predictive spatial and temporal precues and retrocues. The additional inclusion of precues presented before encoding enabled us to compare the effects of attentional orienting to items in VWM with the deployment of perceptual attention to items in view.

### Methods

#### Participants

Twenty-four volunteers (10 women, 14 men; mean age = 24 years; age range: 18–33 years) participated in the experiment for course credit or monetary compensation. All participants had normal or corrected-to-normal visual acuity and colour vision. They were naive to the purpose of the experiment and provided informed written consent. The experimental protocol was approved by the ethics committee of the Department of Psychology at Humboldt-Universität zu Berlin and conducted in accordance with the Declaration of Helsinki (2008).

#### Apparatus and stimuli

Participants sat in a dark room, facing a monitor (ViewPixx/3D monitor, 24-in., 1,920 × 1,080 pixels) at a viewing distance of 53 cm. Stimulus presentation and response collection were controlled using MATLAB (The MathWorks, Natick, MA) and the Psychophysics Toolbox 3 (Brainard, 1997; Kleiner, Brainard, & Pelli, 2007). Participants responded by pressing one of two buttons on a keyboard with their left or right index finger. The assignment of buttons to responses (present or absent) was balanced across participants, randomly assigned for each person and constant throughout experimental sessions.

**Fig. 1:**
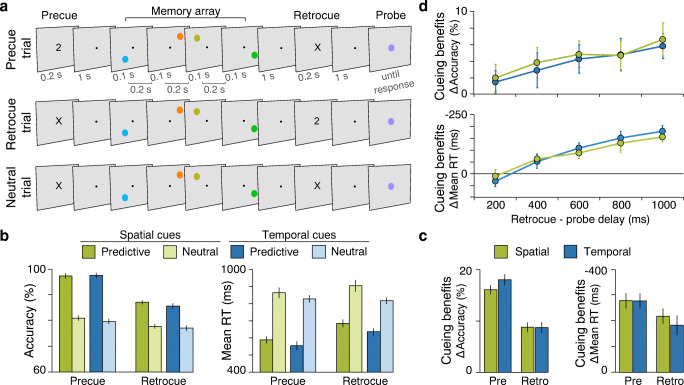
Experiments 1 and 2. **a** Trial procedure for precue, retrocue, and neutral trials of Experiment 1. Each trial started with the presentation of a precue, which was either valid (predictive precue trials) or neutral (neutral precue and all retrocue trials). Valid cues were numbers mapping onto either the spatial or temporal positions (e.g., ‘2’ indicated the orange item in temporal cue blocks and the green item in spatial cue blocks). After an interval of 1s, four memory items were shown sequentially and at different locations. Participants were instructed to memorize their colours. During the retention interval, a retrocue was presented, which was either valid (predictive retrocue trials) or neutral (neutral retrocue and all precue trials). At the end of each trial, a probe appeared centrally, and participants had to indicate if its colour was one of the memorised colours on that trial (present trials) or not (absent trials). **b** Accuracy in percent and mean reaction times for the different cue types (spatial vs. temporal; predictive vs. neutral; precue vs. retrocue) in Experiment 1. Error bars show within-subject standard errors of the means (Cousineau, 2005; Morey, 2008). **c** Cueing benefits (performance with predictive cues minus performance with neutral cues) for the different cue types in Experiment 1. **d** Results of Experiment 2: Cueing benefits for spatial and temporal cues as a function of the delay between retrocue and probe. Error bars show standard errors of the means. (Colour figure online)

The task featured four coloured memory items. Their colours were randomly chosen on each trial from the following set (CIE coordinates x/y; luminance): blue (.093/.347; 48.95 cd/m^2^), green (.051/.720; 47.84 cd/m^2^), orange (.478/.441; 51.85 cd/m^2^), pink (.314/.287; 51.73 cd/m^2^), red (.400/.361; 49.88 cd/m^2^), violet (.232/.285; 52.94 cd/m^2^), and yellow (.338/.502; 49.86 cd/m^2^). The colour of the probe item was either one of the memory item colours (‘present’ trials) or randomly chosen from one of the remaining colours that were not memory item colours on that trial (‘absent’ trials). Memory items were presented at four fixed spatial locations: at 45°, 135°, 225°, and 315° on an imaginary circle at an eccentricity of 5.23 degrees of visual angle (dva). Thus, there was one item in each quadrant of the display. The probe item was presented centrally. All items were disks with a diameter of 1.16 dva. The fixation dot subtended 0.17 dva. Predictive cues were numbers (1–4) that mapped onto either the temporal positions (temporal cues) or spatial locations (spatial cues) of the memory items. More specifically, for temporal cues, the numbers represented the serial position in the memory array (e.g., a ‘2’ would indicate the second item in the sequence—the orange item in Fig. 1a); for spatial cues, numbers represented the four item locations in clockwise order, starting with the top right quadrant (e.g., a ‘2’ would indicate the item in the bottom right quadrant—the green item in Fig. 1a). Neutral cues were an ‘X’. All stimuli were presented on a grey background.

#### Procedure and design

The trial procedure is illustrated in Fig. 1a. Each trial started with the presentation of a precue for 200 ms. In precue trials, the precue was either predictive or neutral. Predictive cues validly indicated one of the to-be-memorised items as the only item, with which the probe item had to be compared. In retrocue trials, the precue was always neutral. Spatial and temporal cues were physically identical, but predictive cues mapped onto spatial or temporal item positions (see ‘apparatus and stimuli’) in different blocks of trials. After an interval of 1,000 ms, four memory items were presented sequentially (each for 100 ms, with 200 ms interstimulus intervals between items). Participants were instructed to memorize their colours. During the maintenance interval—1,000 ms after the offset of the last memory item—a retrocue was presented for 200 ms. In retrocue trials, the retrocue was either predictive or neutral; in precue trials, the retrocue was always neutral. At the end of each trial, a probe item appeared in the centre of the display and participants had to indicate if the probe item colour was one of the memory item colours in that trial (‘present’ trials) or not (‘absent’ trials). In ‘present’ trials with a predictive cue, the probe item colour was always that of the cued item. The probe item was present until response (or up to 10 s), but participants were encouraged to respond both as accurately and quickly as possible. The next trial started after a 1,000 ms intertrial interval (500 ms blank display followed by 500 ms with the fixation dot to signal the beginning of the new trial). Participants were instructed to maintain fixation during the trials.

The experiment consisted of 1,152 trials in total, equally divided among cued dimension (space vs. time), cue timing (precue vs. retrocue) and cue validity (predictive vs. neutral) conditions. In each condition, the probe item colour was ‘present’ in half of all trials, and ‘absent’ in the other half. All item positions were cued and probed equally often. The experiment was conducted in two sessions on separate days (on average 5 days between sessions). Each session consisted of 18 blocks of 32 trials. Cued dimension was varied across sessions (order counterbalanced across participants) and cue timing across blocks of trials (change after the first half of each session). The order of pre- and retrocues was counterbalanced across participants, but constant across sessions. Cue validity (predictive vs. neutral) was randomly drawn on a trial-to-trial basis. Between blocks, participants had the opportunity to take a break.

#### Data analysis

We excluded reaction time outliers (±2.5 *SD* from individual mean RT; 2.2% of all trials; for all experiments, the results based on the complete datasets, including reaction time outliers, are reported in the Supplementary Material) and trials, in which participants failed to respond within the maximum response time (0.03% of all trials) from the analyses. We analyzed accuracy in percent and mean reaction times of correct responses. We further computed the sensitivity to detect a change (*d'*), for which we obtained the same pattern of results as for accuracy in all experiments. For the sake of brevity, we report these results in the Supplementary Material.

Individual accuracy and reaction time measures were submitted to three-way repeated-measures analyses of variance (ANOVAs), with the factors cued dimension, cue timing, and cue validity. For nonsignificant effects of interest (e.g., when temporal and spatial cueing benefits were found not to differ), we additionally computed Bayes Factors indicating the evidence in support of the null hypothesis over the alternative hypothesis (BF_01_) using the default settings of JASP (Version 0.9.1; JASP Team, 2020).

### Results

Figure 1b shows accuracy and reaction times for the different cues. Performance was better with predictive than with neutral cues, accuracy: *F*(1, 23) = 159.17, *p* <.001, partial η^2^ = .874; RT: *F*(1, 23) = 68.343, *p* < .001, partial η^2^ = .748, and all cues types yielded significant cueing benefits (predictive minus neutral; see Fig. 1c; *t* tests against zero, all *p*s < .001, Bonferroni–Holm corrected for multiple comparisons). Precues were more effective than retrocues, as revealed by an interaction of cue timing and cue validity, accuracy: *F*(1, 23) = 36.27, *p* <.001, partial η^2^ = .612; RT: *F*(1, 23) = 19.60, *p* <.001, partial η^2^ = .46. Performance was also overall better with precues than with retrocues, accuracy: *F*(1, 23) = 120.67, *p* < .001, partial η^2^ = .840; RT: *F*(1, 23) = 6.94, *p* = .015, partial η^2^ = .232. This effect, however, was driven by the selective improvement with predictive cues, as performance was at approximately the same level for neutral-cue trials interleaved with different cue types. In terms of reaction times, there was also an effect of cued dimension, *F*(1, 23) = 4.56, *p* = .044, partial η^2^ = .165, with faster reaction times in blocks with temporal cues (710 ms ± 30 ms; Mean ± *SEM*) than in blocks with spatial cues (761 ms ± 42 ms).

Crucially, we found no interaction between cued dimension and cue validity, accuracy: *F*(1, 23) = 0.47, *p* = .499; RT: *F*(1, 23) = 1.42, *p* = .245. In fact, spatial and temporal cues brought about highly similar cueing benefits (see Fig. 1c). Given that accuracy for predictive precues was near ceiling, the comparison of spatial and temporal cueing benefits in precue-trials may not be particularly informative, *t*(23) = 1.33, *p* = .396, BF_01_ = 2.15. However, cueing benefits did not differ for precues in terms of reaction times, *t*(23) = 0.05, *p* = .963, BF_01_ = 4.65, or for retrocues, accuracy: *t*(23) = 0.50, *p* = .625, BF_01_ = 4.17; RT: *t*(23) = 1.46, *p* = .314, BF_01_ = 1.82, either. Thus, spatial and temporal position could be used equally well to prioritise items for encoding or during maintenance. None of the other interactions were significant.

## Experiment 2

In Experiment 1, temporal position information was found to be as effective as spatial location in guiding both external attention to items in the memory array or internal attention in VWM. It is conceivable, however, that participants still adopted a spatial strategy to utilize temporal cues. As all items had unique spatial and temporal coordinates in this task, participants could have used temporal information only for the purpose of retrieving the spatial location of the cued item (see also Heuer & Schubö, 2016a; Pertzov et al., 2013). This would be predicted by models assuming that different features of an object are bound via their shared position in space (e.g., Pertzov & Husain, 2014; Rajsic & Wilson, 2014; Schneegans & Bays, 2017; Treisman & Zhang, 2006): In the present task, colour and serial position would accordingly each be bound to spatial location, but not directly to each other. Having to take a ‘detour’ via location before being able to deploy (spatial) attention can be assumed to require more time. In this scenario, it should thus take longer to make use of temporal cues. In Experiment 2, therefore, we varied the interval between retrocue and probe item to delineate the time course of attentional orienting based on temporal position versus spatial location. To the extent that the spatial detour requires time, an equivalent time course would invalidate the idea that the performance benefit brought about by temporal cues also relies on spatial attention.

### Methods

Unless stated otherwise, the methods of Experiment 2 were the same of those of Experiment 1.

#### Participants

Twenty-four volunteers (18 women, six men; mean age = 23 years; age range: 18–33 years) participated in the experiment; one of them had also participated in Experiment 1.

#### Procedure and design

As there were no precues, each trial started with the presentation of the memory array. The delay between retrocue and probe item was varied on a trial-to-trial basis from 200 ms to 1,000 ms, in steps of 200 ms.

The experiment consisted of 960 trials, organized in 20 blocks of 48 trials each. Trials were equally divided among cued dimension (space vs. time), cue validity (predictive vs. neutral), and retrocue-probe delay (200-1,000 ms). Cued dimension changed after the first half of the experiment; the order of cued dimensions was counterbalanced across participants.

#### Data analysis

We excluded reaction time outliers (±2.5 *SD* from individual mean RT; 2.4% of all trials) and trials in which participants failed to respond within the maximum response time (0.03% of all trials) from the analyses. Individual cueing benefits were submitted to two-way repeated-measures ANOVAs, with the factors cued dimension and retrocue-probe delay.

### Results

Cueing benefits increased with the delay between retrocue and probe (see Fig. 1d). The overall effect of delay was particularly pronounced in terms of reaction times, *F*(4, 92) = 23.97, *p* <.001, partial η^2^ = .51, and just failed to reach significance for accuracy, *F*(4, 92) = 2.372, *p* = .058, partial η^2^ = .093. Importantly, there was neither an effect of cued dimension, accuracy: *F*(1, 23) = 0.19, *p* = .669, BF_01_ = 6.25; RT: *F*(1, 23) = 0.21, *p* = .651, BF_01_ = 6.42, nor an interaction of cued dimension and delay, accuracy: *F*(4, 92) = 0.04, *p* = .997, BF_01_ = 10.43; RT: *F*(4, 92) = 0.70, *p* = .596, BF_01_ = 2.05. Thus, the time courses of attentional orienting were equivalent independently of whether it was based on temporal or spatial position. This result renders the idea that temporal information was only used to retrieve the location of the cued item and deploy spatial attention a highly unlikely scenario.

## Experiment 3

In Experiment 3, we tested temporal and spatial retrocues with different retrieval contexts. Probe items were presented along with three placeholder items—either sequentially and at different locations (as at encoding; spatiotemporal retrieval context), simultaneously and at different locations (spatial retrieval context), or sequentially and at the same location (temporal retrieval context). This manipulation served two purposes. First, it was another way to test if temporal cues are utilized via a spatial mechanism. If that were the case, both spatial and temporal cues should yield larger benefits when the retrieval cue is also spatial, as compared to purely temporal retrieval contexts. Second, the variation of retrieval contexts allowed us to determine whether the orienting of spatial or temporal attention strengthened the binding between the item and its position along the cued dimension. Item-context bindings are critical elements of some theoretical conceptualizations of VWM (Oberauer, 2009; Oberauer & Lin, 2016), and their strengthening is one of several ways in which retrocues may improve memory (e.g., Rerko & Oberauer, 2013; Souza & Oberauer, 2016). In the present task, strengthened bindings between colours and the cued context dimension can be assumed to facilitate retrieval via the cued dimension, which would selectively modulate cueing benefits: Temporal cueing benefits should be larger with temporal retrieval contexts and spatial cueing benefits should be larger with spatial retrieval contexts.

### Methods

Unless stated otherwise, the methods of Experiment 3 were the same as those of Experiment 1.

#### Participants

Twenty-four volunteers participated in the experiment; two of them had also participated in Experiment 1, five in Experiment 2, and one had participated in both Experiments 1 and 2. The data from one participant had to be excluded because performance did not exceed chance level. All analyses were performed on the remaining twenty-three participants (17 women, six men; mean age = 25 years; age range: 18–34 years).

#### Apparatus and stimuli

The enlarged fixation dot presented during response time subtended 0.23 dva.

#### Procedure and design

**Fig. 2:**
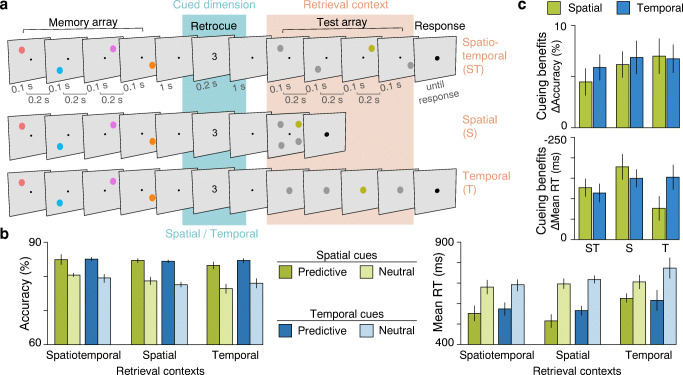
Experiment 3. **a** Trial procedure for the different retrieval context conditions (highlighted in orange): The availability of spatial and temporal information at retrieval was varied by presenting items sequentially and at different locations, as in the memory array (spatiotemporal), simultaneously and at different locations (spatial), or sequentially and at the same location (temporal). Only the probe item was coloured; the remaining three items were grey placeholder items. Participants had to indicate if the colour of the probe item was the same as that of the respective memory item at the same spatial and/or temporal position. Valid retrocues consisted of numbers mapping onto the spatial or temporal positions (varied across blocks of trials). **b** Accuracy in percent and mean reaction times for the different retrieval contexts and retrocues types. Error bars show within-subject standard errors of the means. **c** Cueing benefits (performance with predictive cues minus performance with neutral cues) for the different retrieval contexts and cue types. Error bars show standard errors of the means. (Colour figure online)

The trial procedure is illustrated in Fig. 2a. Each trial started with the sequential presentation of the memory items, followed by a retrocue (spatial vs. temporal and predictive vs. neutral) during the maintenance interval. Unlike in the previous experiments, we additionally manipulated the retrieval context. At the end of each trial, a test array of four items was presented: three were grey placeholder items, one was the probe item. Participants had to judge whether the colour of the probe item was the same as that of the respective memory item, or if it had changed. They were informed that item colours never switched positions—if there was a change, it was to a new colour. We varied the availability of spatial and temporal contexts by presenting items sequentially and at different locations (spatiotemporal), simultaneously and at different locations (spatial), or sequentially and at the same central location (temporal). After the last test item, the fixation dot was enlarged to signal the onset of response time (present until response or for a maximum of 10 s).

The experiment consisted of 960 trials (24 blocks of 40 trials each), equally divided among cued dimension (spatial vs. temporal), retrieval context (spatiotemporal vs. spatial vs. temporal) and cue validity (predictive vs. neutral) conditions. Cued dimension changed after the first half of the experiment (order balanced across participants), retrieval context changed after each four blocks of trials (order balanced across participants but constant across cued dimensions—i.e., the same in both halves of the experiment), and cue validity varied randomly on a trial-to-trial basis.

#### Data analysis

We excluded reaction time outliers (±2.5 *SD* from individual mean RT; 2.6% of all trials) and trials, in which participants failed to respond within the maximum response time (0.04% of all trials) from the analyses. Individual measures were submitted to three-way repeated-measures ANOVAs, with the factors cued dimension (space vs. time), retrieval context (spatiotemporal vs. spatial vs. temporal), and cue validity (predictive vs. neutral).

### Results

Figure 2b shows accuracy and reaction times as a function of retrieval context, cued dimension, and cue validity; Fig. 2c directly shows the corresponding cueing benefits. Overall, performance was better with predictive cues than with neutral cues, accuracy: *F*(1, 22) = 56.65, *p* < .001, partial η^2^ = .720; RT: *F*(1, 22) = 71.96, *p* < .001, partial η^2^ = .766. In fact, all different combinations of retrieval context and cued dimension conditions yielded significant cueing benefits, accuracy: all *p*s < .001; RT: *t*(22) = −2.54, *p* = .009, with temporal retrieval context and spatial cues, all others *p*s <.001; Bonferroni–Holm corrected for multiple comparisons.

There were no main effects of cued dimension, accuracy: *F*(1, 22) = 0.04, *p* = .835, BF_01_ = 7.29; RT: *F*(1, 22) = 0.25, *p* = .62, BF_01_ = 4.72; or retrieval context, accuracy: *F*(2, 44) = 2.52, *p* = .09, BF_01_ = 5.44; RT: *F*(2, 44) = 1.72, *p* = .191, BF_01_ = 2.39. Retrieval context seemed to have a larger effect on performance in neutral cue trials than in valid cue trials, at least in terms of accuracy—while accuracy in neutral trials was affected by retrieval context, *F*(2, 44) = 3.35, *p* = .044, partial η^2^ = .132, replicating previous findings (Heuer & Rolfs, 2021), accuracy in valid trials was not, *F*(2, 44) = 0.44, *p* = .647. Critically, however, none of the interactions reached significance. Paired comparisons of spatial and temporal cueing benefits for each retrieval context condition (Fig. 2c) confirmed that cues relying on either dimension were equally effective irrespective of the availability of spatial or temporal context information at retrieval, in terms of both accuracy, spatiotemporal: *t*(22) = 0.78, *p* = .443, BF_01_ = 3.47; spatial: *t*(22) = 0.06, *p* = .949, BF_01_ = 4.56; temporal: *t*(22) = 0.14, *p* = .88, BF_01_ = 4.53, and reaction time, spatiotemporal: *t*(22) = 0.69, *p* = .499, BF_01_ = 3.69; spatial: *t*(22) = 0.97, *p* = .345, BF_01_ = 3.02; temporal: *t*(22) = 1.63, *p* = .118, BF_01_ = 1.46.

For one, this pattern further invalidates the idea that temporal cues relied on a spatial mechanism: Temporal cueing benefits did not differ between spatial and temporal retrieval contexts, accuracy: *t*(22) = 0.06, *p* = .953, BF_01_ = 4.57; RT: *t*(22) = 0.10, *p* = .925, BF_01_ = 4.55, and neither did spatial cueing benefits in terms of accuracy, *t*(22) = 0.02, *p* = .983, BF_01_ = 4.57. These findings are also inconsistent with the hypothesis that retrocueing strengthened item-context bindings, which predicts better performance when the retrieval cue dimension is congruent with the cued dimension (e.g., temporal cue and temporal retrieval context). Only spatial cueing benefits in terms of reaction time were larger with spatial than with temporal retrieval contexts, *t*(22) = 2.40, *p* = .025, *d* = 0.5.

## General discussion

Our visual environment unfolds over time. Often, this occurs in a predictable manner: Recurring temporal structures give rise to expectations that shape not only perception (Nobre & van Ede, 2018) but also contribute to an effective and efficient use of limited VWM resources by dynamically prioritizing memory contents at the time they are expected to be needed (Jin et al., 2019; van Ede et al., 2017). Sometimes, however, the timing of visual events only renders them relevant in retrospect. Our findings demonstrate that in such a scenario, attention can be oriented towards items held in VWM based on their temporal position as effectively as based on their spatial position: We found equivalent retrocueing benefits across three experiments, different cue-probe intervals and irrespective of whether spatial or temporal position was used as retrieval cue.

As it is widely accepted that space holds a special status in VWM, it is no trivial observation that bindings with either dimension can be utilized equally well for prioritizing specific items. The architecture of VWM has often been conceptualized as location-based (e.g., Schneegans & Bays, 2017; Treisman & Zhang, 2006), meaning that nonspatial features of an object are each bound to its spatial location and thereby only indirectly to each other. Importantly, our findings are incompatible with the idea that temporal cues were only used to retrieve information about an item’s spatial position and thus relied on spatial attention just as much as spatial cues. If that were the case, temporal cues would likely have required more time to be utilised than spatial cues and their retrieval would have been facilitated by spatial retrieval cues. Instead, our findings indicate that the items were directly bound to temporal position. This adds to an emerging picture of time (e.g., ordinal position) playing a similar functional role as space in VWM, providing a context or index to which non-spatiotemporal surface features (e.g., colour or orientation) are bound (see also Schneegans & Bays, 2018). For instance, patterns of binding errors were found to be consistent with a model in which surface features are bound via either spatial or temporal position when items were presented at different locations and sequentially (Schneegans et al., 2020). Moreover, we have recently shown that both spatial and temporal properties are incidentally encoded and functionally relevant, even when they are not required for the task, so that memory is impaired when the distinctive spatial or temporal information is not available at retrieval (Heuer & Rolfs, 2021). Notably, temporal context was, under certain conditions, even more important as a reference frame than spatial context.

A strengthening of item-context bindings has been suggested as one of several non-mutually exclusive mechanisms that may underlie retrocueing benefits (e.g., Rerko & Oberauer, 2013; Souza & Oberauer, 2016). We found no evidence, however, that strengthened item-context bindings contributed to performance benefits in the present task: Cueing an item with a given dimension (e.g., temporal cue) did not selectively facilitate retrieval via the same dimension (e.g., temporal retrieval context) in Experiment 3. It is conceivable that the 100% valid cues encouraged participants to remove the uncued items from memory (Kuo et al., 2012; Souza et al., 2014), essentially reducing memory load to one item. Given that items are likely bound to *relative* positions defined by inter-item relations (e.g., Hollingworth, 2007; Jiang et al., 2000; Treisman & Zhang, 2006), the importance of item-context bindings and thus of retrieval context may accordingly have been diminished in predictive trials. Indeed, this notion is supported by the finding that the manipulation of retrieval context only affected performance in neutral trials.

In summary, we have shown that temporal properties contribute to a flexible (re-)prioritization of visual information held in working memory by drawing attention to representations linked to specific points in time—even when these are only rendered relevant after they have passed. Attentional orienting based on temporal position occurs as directly, swiftly and effectively as orienting based on spatial position, highlighting the potential functional equivalence of the spatial and temporal dimensions in VWM.

## Supplementary Information

ESM 1 (DOCX 21 kb)
