# Supplementary material for: A direct comparison of attentional orienting to spatial and temporal positions in visual working memory
Source: Psychon Bull Rev. 2021 Jul 21;29(1):182–90. doi: 10.3758/s13423-021-01972-3 (PMC8858307; doi:10.3758/s13423-021-01972-3)
Supplement: Supplementary file 1 — (DOCX 21 kb) [file 13423_2021_1972_MOESM1_ESM.docx]

**Supplementary Material**

A direct comparison of attentional orienting to spatial and temporal positions

in visual working memory

Anna Heuer and Martin Rolfs

**Supplementary methods**

Sensitivity was calculated as d’ = z(hit rate) – z(false alarm rate). Rates of 0 were replaced with 0.5/n and rates of 1 were replaced with (n–0.5)/n, where n is the number of change or no-change trials, respectively (Stanislaw & Todorov, 1999).

**Supplementary results**

**Sensitivity to detect a change (d’)**

For the sake of brevity, we only report accuracy in percent and mean reaction times in the main text. We obtained the same pattern of results for the sensitivity to detect a change (d’).

Experiment 1

Sensitivity was higher with predictive than with neutral cues (Mean ± SEM; *predictive*: 3.24 ± 0.09; *neutral*: 1.81 ± 0.13; F_(1,23)_ = 497.23, p < .001, partial η^2^ = .956) and all cues types yielded significant cueing benefits (predictive minus neutral; *spatial precue*: 2.04 ± 0.15; *temporal precue*: 2.25 ± 0.13; *spatial retrocue*: 0.71 ± 0.08; *temporal retrocue*: 0.71 ± 0.10; t-tests against zero, all p < .001, Bonferroni-Holm corrected for multiple comparisons). Precues were more effective than retrocues, as revealed by an interaction of cue timing and cue validity (F_(1,23)_ = 112.00, p <.001, partial η^2^ = .830). Sensitivity was also overall higher with pre- than with retrocues (F_(1,23)_ = 245.18, p < .001, partial η^2^ = .914); this effect was driven by the selective improvement with predictive cues.

Crucially, we found no interaction between cued dimension and cue validity (F_(1,23)_ = 1.17, p = .292, BF_01_ = 6.14). In fact, spatial and temporal cues brought about highly similar cueing benefits that did not differ for precues (t_(23)_ = 1.29, p = .210, BF_01_ = 2.23) or retrocues (t_(23)_ = -0.003, p = .997, BF_01_ = 4.66). There was no main effect of cued dimension (F_(1,23)_ = 0.48, p = .496, BF_01_ = 5.73) and none of the other interactions were significant, either.

Experiment 2

Cueing benefits increased with delay duration (*200 ms*: 0.20 ± 0.09; *400 ms*: 0.29 ± 0.11; *600 ms*: 0.38 ± 0.08; *800 ms*: 0.45 ± 0.11; *1000 ms*: 0.50 ± 0.11), but the effect of delay failed to reach significance (F_(4,92)_ = 2.00, p = 0.101, partial η^2^ = .080). Importantly, there was neither an effect of cued dimension (F_(1,23)_ = 0.41, p = .530, BF_01_ = 5.14) nor an interaction of cued dimension and delay (F_(4,92)_ = 0.13, p = .969, BF_01_ = 18.78).

Experiment 3

Sensitivity was higher with predictive cues than with neutral cues (*predictive*: 2.30 ± 0.69; *neutral*: 1.79 ± 0.70; F_(1,22)_ = 55.34, p < .001, partial η^2^ = .716). In fact, all different combinations of retrieval context and cued dimension conditions yielded significant cueing benefits (*ST, spatial retrocue*: 0.36 ± 0.10, t_(22)_ = 3.62, p = .002; *ST, temporal retrocue*: 0.52 ± 0.13, t_(22)_ = 3.83, p < .001; *S, spatial retrocue:* 0.59 ± 0.100, t_(22)_ = 5.90, p < .001; *S, temporal retrocue*: 0.51 ± 0.13, t_(22)_ = 4.12, p < .001; *T, spatial retrocue*: 0.60 ± 0.15, t_(22)_ = 3.96, p < .001; *T, temporal retrocue*: 0.46 ± 0.12. t_(22)_ = 3.72, p = .002; Bonferroni-Holm corrected for multiple comparisons). There were no main effects of cued dimension (F_(1,22)_ = 0.17, p = .687, BF_01_ = 6.74) or retrieval context (F_(2,44)_ = 1.93, p = .157, BF_01_ = 8.09).

Critically, none of the interactions reached significance. Paired comparisons of spatial and temporal cueing benefits for each retrieval context condition confirmed that cues relying on either dimension were equally effective irrespective of the availability of spatial or temporal context information at retrieval (*ST*: t_(22)_ = 0.82, p = .419, BF_01_ = 3.37; *S*: t_(22)_ = 0.454, p = .654, BF_01_ = 4.16; *T:* t_(22)_ = 0.82, p = .419, BF_01_ = 3.37). Temporal cueing benefits did not differ between spatial and temporal retrieval contexts (t_(22)_ = 0.42, p = .682, BF_01_ = 4.23) and neither did spatial cueing benefits (t_(22)_ = 0.03, p = .978, BF_01_ = 4.57).

**Results based on all trials (no exclusion of reaction time outliers)**

To ensure that the exclusion of reaction time outliers did not bias the results, we confirmed all main analyses using the complete datasets (only trials in which participants failed to respond were excluded).

Experiment 1

Performance was better with predictive than with neutral cues (accuracy: F_(1,23)_ = 174.40, p <.001, partial η^2^ = .883; reaction time: F_(1,23)_ = 76.70, p < .001, partial η^2^ = .769) and all cues types yielded significant cueing benefits (t-tests against zero, all p <.001, Bonferroni-Holm corrected for multiple comparisons). Precues were more effective than retrocues, as revealed by an interaction of cue timing and cue validity (accuracy: F_(1,23)_ = 35.05, p <.001, partial η^2^ = .604; reaction time: F_(1,23)_ = 35.38, p <.001, partial η^2^ = .606). Performance was also overall better with pre- than with retrocues (accuracy: F_(1,23)_ = 122.37, p <.001, partial η^2^ = .842; reaction time: F_(1,23)_ = 5.71, p = .025, partial η^2^ = .199). In terms of reaction times, there was also a main effect of cued dimension (F_(1,23)_ = 4.53, p = .044, partial η^2^ = .165), with faster reaction times in blocks with temporal cues than in blocks with spatial cues.

Crucially, there was no interaction between cued dimension and cue validity (accuracy: F_(1,23)_ = 0.96, p = .339; reaction time: F_(1,23)_ = 1.94, p = .177). Spatial and cueing benefits did not differ for precues (accuracy: t_(23)_ = 1.40, p = .174, BF_01_ = 1.96; reaction time: t_(23)_ = 0.30, p = .769, BF_01_ = 4.47) or retrocues (accuracy: t_(23)_ = 0.18, p = .860, BF_01_ = 4.59; reaction time: t_(23)_ = 1.55, p = .135, BF_01_ = 1.64).

Experiment 2

Cueing benefits increased with delay duration (accuracy: F_(4,92)_ = 2.60, p = .04, partial η^2^ = .102; reaction time: F_(4,92)_ = 22.86, p <.001, partial η^2^ = .498). There was neither an effect of cued dimension (accuracy: F_(1,23)_ = 0.08, p = .780, BF_01_ = 6.54; reaction time: F_(1,23)_ = 0.68, p = .417, BF_01_ = 5.20) nor an interaction of cued dimension and delay (accuracy: F_(4,92)_ = 0.04, p = .997; reaction time: F_(4,92)_ = 0.90, p = .466).

Experiment 3

Overall, performance was better with predictive cues than with neutral cues (accuracy: F_(1,22)_ = 56.69, p < .001, partial η^2^ = .720; reaction time: F_(1,22)_ = 67.50, p <.001, partial η^2^ = .754). There were no main effects of cued dimension (accuracy: F_(1,22)_ = 0.19, p = .667, BF_01_ = 6.60; reaction time: F_(1,22)_ = 0.16, p = .695, BF_01_ = 5.70) or retrieval context (accuracy: F_(2,44)_ = 2.06, p = .14, BF_01_ = 7.39; reaction time: F_(2,44)_ = 1.38, p = .263, BF_01_ = 3.96). None of the interactions reached significance for accuracy. In terms of reaction times, there was an interaction between retrieval context and cue validity (F_(2,44)_ = 3.99, p = .026, partial η^2^ = .154) and a three-way interaction (F_(2,44)_ = 6.44, p = .004, partial η^2^ = .227). Paired comparisons of spatial and temporal cueing benefits for each retrieval context condition confirmed that cues relying on either dimension were equally effective irrespective of the availability of spatial or temporal context information at retrieval, in terms of both accuracy (spatiotemporal: t_(22)_ = 0.85, p = .403, BF_01_ = 3.30; spatial: t_(22)_ = 0.08, p = .940, BF_01_ = 4.56; temporal: t_(22)_ = 0.05, p = .959, BF_01_ = 4.57) and reaction time (spatiotemporal: t_(22)_ = 0.37, p = .716, BF_01_ = 4.30; spatial: t_(22)_ = 1.17, p = .253, BF_01_ = 2.48); the only exception was that responses with a temporal retrieval context were faster in temporal cue blocks than in spatial cue blocks (t_(22)_ = 2.70, p = .013, d = 0.56). This effect, however, was driven by slower response in the neutral trials that were interleaved in temporal cue blocks – reaction times following predictive spatial or temporal cues with temporal retrieval contexts did not differ – and thus cannot be attributed to differences in the efficacy of spatial and temporal cues (see **Fig. 2b**, right panel).

This pattern further invalidates the idea that temporal cues relied on a spatial mechanism: Temporal cueing benefits did not differ between spatial and temporal retrieval contexts (accuracy: t_(22)_ = 0.199, p = .844, BF_01_ = 4.49; reaction time: t_(22)_ = 0.10, p = .922, BF_01_ = 4.55) and neither did spatial cueing benefits in terms of accuracy (t_(22)_ = 0.16, p = .877, BF_01_ = 4.52). These findings are also inconsistent with the hypothesis that retrocueing strengthened item-context bindings, which predicts better performance when the retrieval cue dimension is congruent with the cued dimension. Only spatial cueing benefits in terms of reaction time were larger with spatial than with temporal retrieval contexts (t_(22)_ = 2.99, p = .007, d = 0.62).

**References**

Stanislaw, H. & Todorov N. (1999). Calculation of signal detection theory measures. *Behavior Research Methods, Instruments, & Computers,* *31*, 137-149.
